# Sexually dimorphic tibia shape is linked to natural osteoarthritis in STR/Ort mice

**DOI:** 10.1016/j.joca.2018.03.008

**Published:** 2018-06

**Authors:** B. Javaheri, H. Razi, M. Piles, R. de Souza, Y.-M. Chang, I. Maric-Mur, M. Hopkinson, P.D. Lee, A.A. Pitsillides

**Affiliations:** † Skeletal Biology Group, Comparative Biomedical Sciences, The Royal Veterinary College, Royal College Street, London, NW1 0TU, UK; ‡ Max Planck Institute of Colloids and Interfaces, Department of Biomaterials, Research Campus Golm, 14424, Potsdam, Germany; § Institute for Food and Agriculture Research and Technology, Torre Marimon S/n, 08140, Caldes de Montbui, Barcelona, Spain; ‖ Universidade Federal de Mato Grosso (UFMT), Departamento de Clínica, Cuiabá, Brazil; ¶ Manchester X-Ray Imaging Facility, University of Manchester, Manchester, M13 9PL, UK

**Keywords:** Osteoarthritis, Bone shape, STR/Ort, Pain, Gait

## Abstract

**Objectives:**

Human osteoarthritis (OA) is detected only at late stages. Male STR/Ort mice develop knee OA spontaneously with known longitudinal trajectory, offering scope to identify OA predisposing factors. We exploit the lack of overt OA in female STR/Ort and in both sexes of parental, control CBA mice to explore whether early divergence in tibial bone mass or shape are linked to emergent OA.

**Method:**

We undertook detailed micro-CT comparisons of trabecular and cortical bone, multiple structural/architectural parameters and finite element modelling (FEM) of the tibia from male and female STR/Ort and CBA mice at 8–10 (pre-OA), 18–20 (OA onset) and 40 + weeks (advanced OA) of age.

**Results:**

We found higher trabecular bone mass in female STR/Ort than in either OA-prone male STR/Ort or non-prone CBA mice. Cortical bone, as expected, showed greater cross-sectional area in male than female CBA, which surprisingly was reversed in STR/Ort mice. STR/Ort also exhibited higher cortical bone mass than CBA mice. Our analyses revealed similar tibial ellipticity, yet greater predicted resistance to torsion in male than female CBA mice. In contrast, male STR/Ort exhibited greater ellipticity than both female STR/Ort and CBA mice at specific cortical sites. Longitudinal analysis revealed greater tibia curvature and shape deviations in male STR/Ort mice that coincided with onset and were more pronounced in late OA.

**Conclusion:**

Generalised higher bone mass in STR/Ort mice is more marked in non OA-prone females, but pre-OA divergence in bone shape is restricted to male STR/Ort mice in which OA develops spontaneously.

## Introduction

Osteoarthritis (OA), the commonest arthritic disease, causes pain and limits mobility^1^, ^2^. Major (39–65%) genetic contribution is reported for idiopathic hand and knee OA^3^; other risk factors include obesity and high bone density. Bone's aetiological contribution to OA remains obscure, due partly to the complex, ill-defined links to OA joint pathology. It is proposed that in OA subchondral bone, where turnover can be 20-fold higher than normal, exerts a prominent role^4^. Bone adaptation to altered mechanics may also occur more rapidly than in cartilage, inferring that OA bone changes may simply be detectable earlier^5^. Recent observations of greater OA incidence in individuals with higher systematic bone mineral density [BMD^6^, ^7^;] have led to new questions about how bone density and mass are linked to OA development.

Longitudinal studies have linked higher BMD to raised radiographic OA risk^8^, ^9^. Indeed, several early age-onset, high bone mass (HBM) phenotypes^10^ exhibit increases in both joint replacement rates and non-steroidal anti-inflammatory drug use, implying raised OA risk^11^, ^12^. Hereditary canine OA predisposition in larger rapidly growing breeds^13^ and raised knee OA risk with skeletal misalignment suggest that the OA contribution of HBM is conferred anatomically. Misalignment likely perturbs load transmission/stress distribution, increasing radiographic OA risk, suggesting that bone shape also contributes to OA development^14^, ^15^.

These relationships are difficult to resolve in humans, where late OA detection often allows for only post-mortem bone sample collection^16^. Mouse strains also develop OA spontaneously. Inbred STR/Ort, derived from a cross including CBA mice as a parental strain, show spontaneous histological, biochemical and structural similarities to human OA with a predictable and accelerated time-course^17^, ^18^, ^19^, ^20^, ^21^, ^22^. Male STR/Ort mice show histological cartilage fibrillation principally affecting the medial tibial condyle from ∼16 weeks; severe OA in ∼85% by 35 weeks and up to 100% by 15 months^17^, ^23^, ^24^, ^25^. Whilst the reasons for this OA largely remain obscure^26^, ^27^, ^28^ it is intriguing that female STR/Ort are seemingly protected until 13–15 months of age^19^, ^27^, ^28^.

An elegant study by Stok *et al.*, (2009) using male CBA, as controls, evaluated bone mass and architecture during STR/Ort mouse ageing; found higher trabecular, cortical and subchondral bone mass in male STR/Ort mice^16^. Further studies, using C57BL/6, as controls, described HBM in the femur of 1 month-old STR/Ort mice with shrinkage in the medullary cavity. This was attributed to an osteoclastogenic blockade and enhanced osteoblast activity, which surprisingly was more marked in female than in OA-prone male STR/Ort mice^29^. Uchida *et al.*, (2012) compared BMD and architecture in male and female STR/Ort mice aged 5–35 weeks, reporting that neither age- nor gender-related differences independently explain OA predisposition and timing in males^30^.

We have undertaken systematic cross-sectional examination of tibial bone phenotype in OA-prone male and non-prone female STR/Ort mice and healthy male and female parental, control CBA mice, at specific phases corresponding to pre-OA, OA onset and advanced OA stages. We have evaluated trabecular bone mass in the proximal tibial metaphysis and cortical shape and geometry traits, and predicted load-bearing impact along the entire tibial shaft by finite element modelling (FEM). The intention was two-fold: to identify the extent to which age and gender interact to support HBM in the STR/Ort strain and - on the basis that higher bone mass in female than male STR/Ort mice would be confirmed - examine whether instead, differences in tibia shape might explain gender-related OA links. We have also explored three-way statistical interactions of gender and genotype with age, as the time course for OA development in STR/Ort mice is well established. We have examined many aspects of bone mass and shape as exploration of our hypothesis is not based upon any specific parameter but a group of parameters that collectively describe bone structure.

We confirm that generalised HBM in the STR/Ort strain is indeed more marked in non OA-prone females, and disclose that pre-OA divergence in bone shape restricted only to male STR/Ort mice is a unique feature related to the spontaneous onset of OA in this model.

## Materials and methods

### Animals

CBA (Charles River, UK) and STR/Ort mice (Royal Veterinary College (RVC) London, UK) were housed in polypropylene cages under 12 h light/dark cycle at 21 ± 2°C with free access to rat/mouse one maintenance diet (Special Diet Services, Witham UK) and water *ad libitum*. All procedures complied with UK Animals (Scientific Procedures) Act 1986, were approved by RVC's ethics committee and comply with ARRIVE guidelines^31^. Body weight was recorded (Supplementary Table I).

### Gait analysis

Gait was recorded by a treadmill-based DigiGait™ system (Mouse Specifics, Boston^32^,) and analysed as described^33^. Briefly, male and female STR/Ort mice (n = 31/24 respectively) ran at 17 cm/s (for <30 s) while a video-camera captured ventral images; 5 s segments (>10 consecutive strides). Symmetry indices/ratios, compensation and contralateral fore/hind limb balance were computed^34^; 101 left/right side descriptors were recoded as minimum (primary) and maximum (secondary). Asymmetry measures allow for monitoring of unpredictable left/right OA targeting in STR/Ort limbs. To avoid bias, left/right differences were negated by denoting these as max/min instead (L/R and R/L become additive). Greater symmetry indicates more ‘normal’ gait (proviso that both limbs may be affected equally). Mouse treadmill task non-compliance (inability/unwillingness to complete treadmill task^35^) was recorded.

### X-ray microcomputed tomography (μCT)

Scanning and analysis was performed as described^33^, ^36^, ^37^. Briefly, additional male and female, CBA and STR/Ort mice were sacrificed at either 8–10, 18–20 and 40 weeks-old (*n =* 5 at each age; total 60 mice). Right tibiae were fixed in 4% formaldehyde and stored in 70% EtOH until scanning. Entire tibiae were scanned using Skyscan 1172 (Skyscan, Kontich, Belgium), with X-ray tube at 50 kV and 200 μA, 1600 ms exposure time and 5 μm voxel size. Slices were reconstructed using NRecon1.6, 2D/3D analyses performed using CTAn1.15 + and CTvox3.1 used for colour-coded images of thickness.

### Morphometric trabecular bone analysis

Appearance of the trabecular ‘bridge’ connecting the two primary spongiosa bone ‘islands’ set as reference point for analysis of proximal tibia metaphyseal trabecular bone; 5% of total bone length from this point (towards diaphysis) was utilised for trabecular analysis.

### Whole bone cortical analysis

Whole bone analysis was performed using BoneJ^38^, an ImageJ plugin^39^. Following segmentation, alignment and removal of fibula, a minimum threshold was used in “Slice Geometry” to calculate mass: cross sectional area (CSA), mean thickness (Ct.Th), and shape (second moment of area around minor (*I*_min_) and major axes (*I*_max_), ellipticity and predicted resistance to torsion (*J*)). Calibrated μCT was used to assess cortical tissue mineral density (TMD) across 100 cortical slices at 37% of length.

### Histology and grading of articular cartilage (AC) lesions

Right knees (*n* = 5) from mice which underwent gait analysis were fixed, decalcified, wax-embedded and 6 μm coronal sections cut. Multiple slides (∼10), each containing five sections sampled at 120 μm intervals spanning the entire joint, were stained with Toluidine blue and AC lesion severity scored using an internationally-recognised system^40^, ^41^. Grading in compartments (lateral/medial, tibia/femur) allowed for maximum grade to be assigned in each section, and used to generate an overall ‘average’ maximum grade/group of mice. Mean score for each joint and compartment was produced an overall ‘average’ mean grade.

### Finite element analysis

Local strains were characterized by FEM^42^. Briefly, models (*n* = 1/group) were created in Abaqus 6.14–5 software (Dessault Systemes, Providence, RI). MicroCT images were discretised with multi-resolution volumetric linear tetrahedral mesh elements (∼1.2e6 elements/bone) using ZIBAmira software (Zuse Institute, Berlin, Germany). FEM boundary conditions replicated axial loading condition^43^. Alignment was achieved by defining a longitudinal axis using anatomical landmarks^42^. Contact surfaces at distal tibia were fixed in all degrees of freedom and at proximal tibia, restrained from off-axis movement from loading axis, which was inserted proximally. To isolate the effect of morphology, a similar homogeneous material property was assigned (Young's modulus: 17 GPa and Poisson's ration: 0.3)^42^. Total strain and stress were calculated/element, von Misses stress, absolute maximum principal strain and moment arm (curvature lever arm; distance of tibial centroid to loading axis) was calculated per cross section. Pre-/post-processing was performed using MATLAB (The Mathworks Inc.).

### Statistical analyses

We have reported previously that 7 mice/group is sufficient to reproducibly obtain significant differences for gait analysis^35^ and that 5 mice/group provides sufficient power to find significant differences for CT analysis^36^, ^37^.

### Gait

Fisher's exact test was used to assess drop-out. Principal component analysis was performed to extract variation from multivariate gait data and to express this as a set of new uncorrelated variables (principal components, PC), using the function prcomp() (“R”; R Foundation for Statistical Computing, Austria). Linear mixed effects models that account for fixed effects of gender, linear and quadratic polynomials of age and their interactions were employed to assess differences in PCs. Choice of quadratic polynomials of age to describe the longitudinal patterns of gait components was based on the depicted scatter plots. Random effects included intercept, linear and quadratic polynomials of age nested within mice. Normality and homogeneity of variance of residuals were assessed visually (histogram and scatter plot of residuals vs fitted values).

### Trabecular bone

A Shapiro–Wilk normality test (GraphPad Software, CA) was performed on all datasets; all exhibited *P*-values >0.05. A three-way ANOVA univariate linear model was used to analyse how fixed factors (age, genotype and gender) and two-way (genotype*gender, genotype*age, gender*age) and three-way interaction (genotype*gender*age) affected dependent parameters (SPSS Statistics). Proportion of variation explained by the model (*R*^2^) was reported.

### Cortical bone

Graphs were generated using “R”. Three-way ANOVA was used to assess effect of gender, genotype and age at each percentile. Normality and homogeneity of variance of the residuals were checked using Shapiro–Wilk and Bartlett's tests respectively. Data were expressed as mean with 95% confidence interval (CI).

## Results

### Sexually dimorphic OA development in STR/Ort mice is linked to longitudinal gait asymmetry

Our data reveal that no females, but 48% of male STR/Ort mice ‘dropped out’ of the treadmill task (*P* < 0.0001). PC1-PC4 explained 24, 15, 10 and 7% of total gait variation. Differences in longitudinal patterns between male and female STR/Ort (PC1/PC3, Fig. 1(A)) show that linear and quadratic polynomials of age or their interactions with gender had significant effects on PC1, PC3 and PC4 (*P* = 0.007, <0.0001 and 0.01, respectively). Gender had impact on PC1 through interaction with age (*P* = 0.007 for linear and *P* = 0.021 for quadratic polynomial) and also on PC3 via interaction with quadratic polynomial of age (*P* = 0.022). Contribution of gait parameters to PC1-PC4 (illustrated as heatmaps) show that PC1 and PC3, but not PC2 or PC4, are significantly modified between male and female STR/Ort mice; main contributors are stride length and frequency, swing, stance, and propel times, as well as L/R asymmetry indices/ratios for propel, stance, stride frequency and length (Supplementary Fig. 1(A)–(B)). Parameter estimates (95% confidence interval) of fixed effects and variances for the random effects and residual for the first four PCs using linear mixed effects models is provided (Supplementary Table II).

**Fig. 1:**
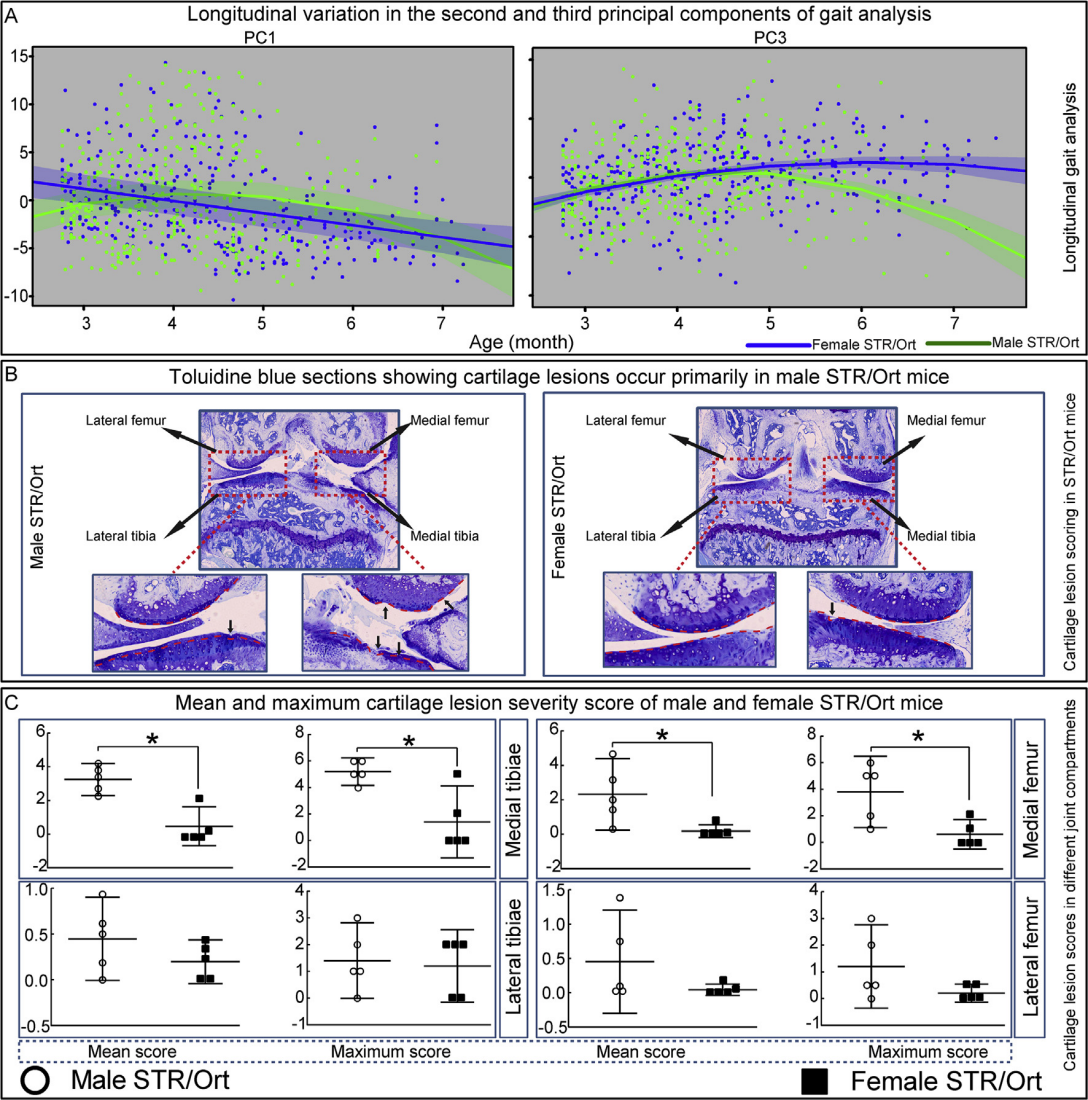
**Sexually dimorphic Osteoarthritis (OA) development and progression is linked to development of longitudinal gait asymmetry and indices of spontaneous OA in STR/Ort mice.** (*A***)** Linear graphs depict principal component distribution and differences in gait patterns of male (green) and female (blue) STR/Ort mice for PC1 and PC3 longitudinally; left/right differences were negated by referring to these as max/min ensuring that both L/R and R/L asymmetries will be additive. (*B*) Cartilage lesion scores in different joint compartments of male and female STR/Ort mice. Mean and maximum with 95% confidence interval (CI) lesion severity scores in each compartment of male (circle) and female (square) STR/Ort joints. (*C***)** Lower and higher power toluidine blue stained sections of joints from male and female STR/Ort mice showing locations of naturally occurring lesions in the articular cartilage (AC) of the lateral femur compartment of the tibiofemoral joint. For gait analysis group sizes were *n* = 31 and *n* = 24 for male and female STR/Ort mice, respectively. For cartilage lesion scoring group sizes were *n* = 5 for male and female STR/Ort mice.

To verify that these sexually-dimorphic gait anomalies were linked to OA severity, we scored AC lesions in STR/Ort mice (*n* = 5/group). In keeping with previous studies^19^, ^27^, ^28^, we find that OA predominates across the joint's medial aspect and mean/maximum scores were significantly higher in male STR/Ort mice [Fig. 1(B)–(C)], indicating a link between greater gait asymmetry, which is arrhythmic and turbulent, and OA severity in male STR/Ort mice.

### Female STR/Ort mice have higher bone mass than OA-prone males and parental CBA mice

Micro-CT showed that age and gender were significant factors in tibia length (*P* ≤ 0.001 and < 0.05 respectively; Table I). To explore if age and gender interact to support the HBM STR/Ort phenotype^29^, analyses focused on trabecular proximal metaphysis, where age was not a significant determinant of BV/TV, whereas genotype and gender both contributed significantly (*P* ≤ 0.001 and ≤ 0.01 respectively; Table I). Female STR/Ort exhibited higher BV/TV than male STR/Ort at all ages [Fig. 2(A)–(B)]. In contrast, male and female CBA exhibited no gender-related difference in BV/TV, which was markedly lower than age-/gender-matched STR/Ort mice. Trabecular number was greater in 40 week-old female STR/Ort than age-matched male STR/Ort [Fig. 2(A)] and virtually identical trends were found at younger ages, suggesting greater retention of trabeculae in female STR/Ort mice at advanced age.

**Fig. 2:**
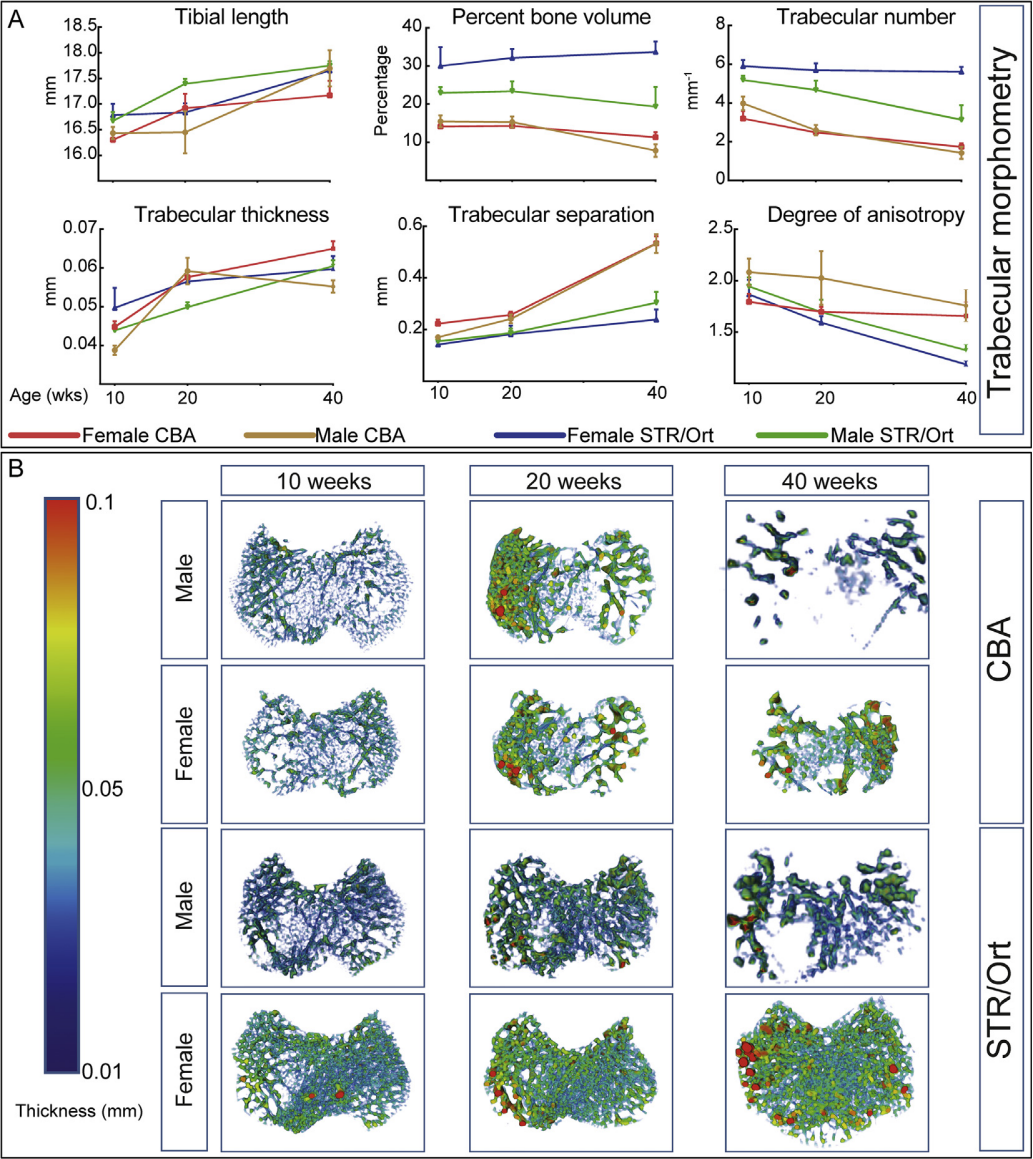
**Analysis of trabecular bone phenotype at the tibial metaphysis of male and female STR/Ort mice.** (*A*) Percent bone volume, trabecular number, thickness and separation. (*B*) Trabecular thickness heatmap of male and female CBA and STR/Ort mice. Line graphs represent means with 95% CI. Group sizes were *n* = 5 for male and female CBA and STR/Ort mice.

**Table I:** **Tibial bone parameters in male and female and STR/Ort and CBA mice at 10, 20 and 40 weeks of age detailing overall effect of age, genotype, gender and their interactions**. Bone parameters include bone length, trabecular (percent bone volume, trabecular number, thickness, separation and degree of anisotropy) and cortical (BMD and total porosity). Group sizes were *n* = 5 for male and female CBA and STR/Ort mice. P > 0.05 was considered non-significant (NS)

Parameter	Genotype	Gender	Age	Genotype* Gender	Genotype* Age	Gender* Age	Genotype* Gender* Age	R-squared
**Bone length (mm)**	≤0.05	NS	≤0.001	NS	NS	NS	NS	0.492
**Trabecular**								
Percent bone volume (%)	≤0.001	≤0.01	NS	≤0.05	NS	NS	NS	0.685
Trabecular number (mm^−1^)	≤0.001	≤0.05	≤0.001	≤0.001	NS	≤0.05	NS	0.817
Trabecular thickness (mm)	NS	≤0.01	≤0.001	NS	≤0.05	NS	≤0.05	0.685
Trabecular separation (mm^−1^)	≤0.001	NS	≤0.001	NS	≤0.001	NS	NS	0.868
Degree of anisotropy	≤0.01	≤0.05	≤0.001	NS	≤0.05	NS	NS	0.529
**Cortical**								
Cortical BMD (g.cm^−3^)	NS	≤0.01	≤0.001	≤0.05	≤0.001	NS	≤0.001	0.857
Total porosity (%)	≤0.001	NS	<0.05	<0.01	<0.05	NS	NS	0.599

CBA mice showed no gender-related divergence in trabecular bone, but lower trabecular number than STR/Ort mice at all ages. This indicates that age, genotype and gender are all significant determinants of trabecular architecture (*P* ≤ 0.001, ≤0.001 and ≤ 0.05, respectively; Table I). Trabecular separation was significantly altered by age and genotype and, thickness altered by age and gender with additional genotype and age interaction (*P* ≤ 0.001, ≤0.01 and ≤ 0.001 respectively; Table I). Male and female STR/Ort showed more marked age-related decline in degree of anisotropy than CBA mice [Fig. 2(A)], with greater age/gender input to trabecular HBM in females than OA-prone males of this strain.

### Genotype-related divergence in cortical thickness is amplified by ageing of STR/Ort mice

Proximodistal analysis showed significant widespread effects of genotype and age, with no major interaction influencing Ct.Th [Fig. 3(A)–(D)]. Further scrutiny disclosed markedly higher Ct.Th in proximal regions in male compared to female STR/Ort mice, which was exaggerated with age [Fig. 3(B)–(C)]; 40 week-old STR/Ort mice diverging markedly in proximal (10–30%) and distal (70–80%) regions. Longitudinal comparison [Fig. 3(B)–(C)] revealed only modest age-related Ct.Th changes in male and female CBA mice. In contrast, significant age-related increases in tibial Ct.Th were found in STR/Ort mice, most prominently in older females. These data reveal that genotype, age and gender are significant determinants of Ct.Th, with significant interactions of genotype and gender and genotype and age in many locations [Fig. 3(D)]. Significant three-way interactions of genotype, gender and age was detected, suggesting that individual contribution of each factor is difficult to separate and that contribution of each factor is dependent upon interaction with the other two; these interactions are, however, only evident in small regions towards the distal tibia (∼70%, ∼85%).

**Fig. 3:**
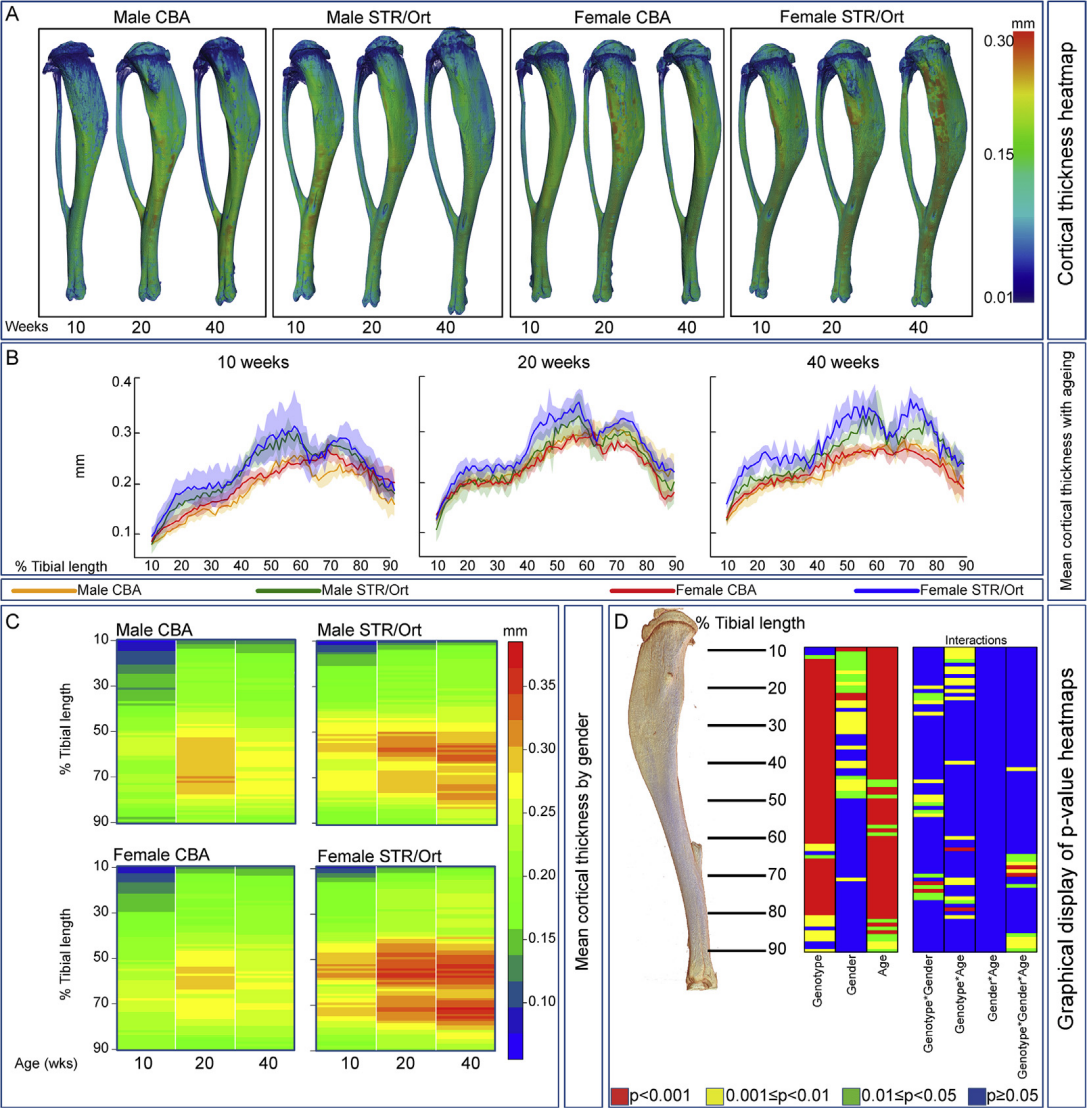
**Analysis of mean thickness along the entire length of the tibia.** (*A*) Representative 3D Micro-CT colour-coded images of tibial cortical bone thickness. (*B*) Mean cortical thickness in male and female CBA (brown and red, respectively) and STR/Ort (green and blue, respectively) mice at 10, 20 and 40 weeks of age. (*C*) ‘Heat map’ representation of identical data-set (red-blue colour scale depicts average mean thickness) for male and female CBA (left) and male and female STR/Ort (right) enabling ready comparison (at each percentile of length) between 10, 20 and 40 weeks of age. (*D*) Statistical significance of differences in mean cortical thickness along the entire tibial shaft, represented as a heat map. The contributions of genotype (CBA vs STR/Ort), gender, age (10, 20 and 40 weeks), and their interactions at locations from 10 to 90% of tibial length are illustrated. Red *P* < 0.001, yellow 0.001 ≤ *P* < 0.01, green 0.01 ≤ *P* < 0.05 and blue *P* ≥ 0.05. Line graphs represent means with 95% CI. Group sizes were *n* = 5 for male and female CBA and STR/Ort mice.

### Gender-related divergence in cortical bone mineralisation density (BMD) at 10 weeks

Cortical BMD also showed significant three-way interactions of genotype, gender and age (Table I). Female STR/Ort and both male/female CBA mice nonetheless showed age-related increases in cortical BMD which, strikingly, were absent in male STR/Ort. Indeed, BMD in STR/Ort and CBA males showed sexually-dimorphic deviation from equivalent females at 10 weeks. Thus, female STR/Ort and both female and male CBA mice show similar age-related changes in cortical BMD but markedly different trajectories were detected before OA onset in male STR/Ort mice.

### Female STR/Ort have greater CSA but males exhibit distinct regional, structural bias

To test whether cortical shape/geometry traits are OA-linked, we examined matched tibial sites [Fig. 4(A)–(C)] to find that genotype significantly affects CSA along almost the entire bone (10–80%). Gender and age affected CSA from mid-shaft to distal portions (30–90%), indicating strong interaction of genotype and gender. Significant three-way interactions of genotype, gender and age was evident in only a small distal section (∼80%), indicating that distinct contribution of age, gender and genotype are difficult to decipher.

**Fig. 4:**
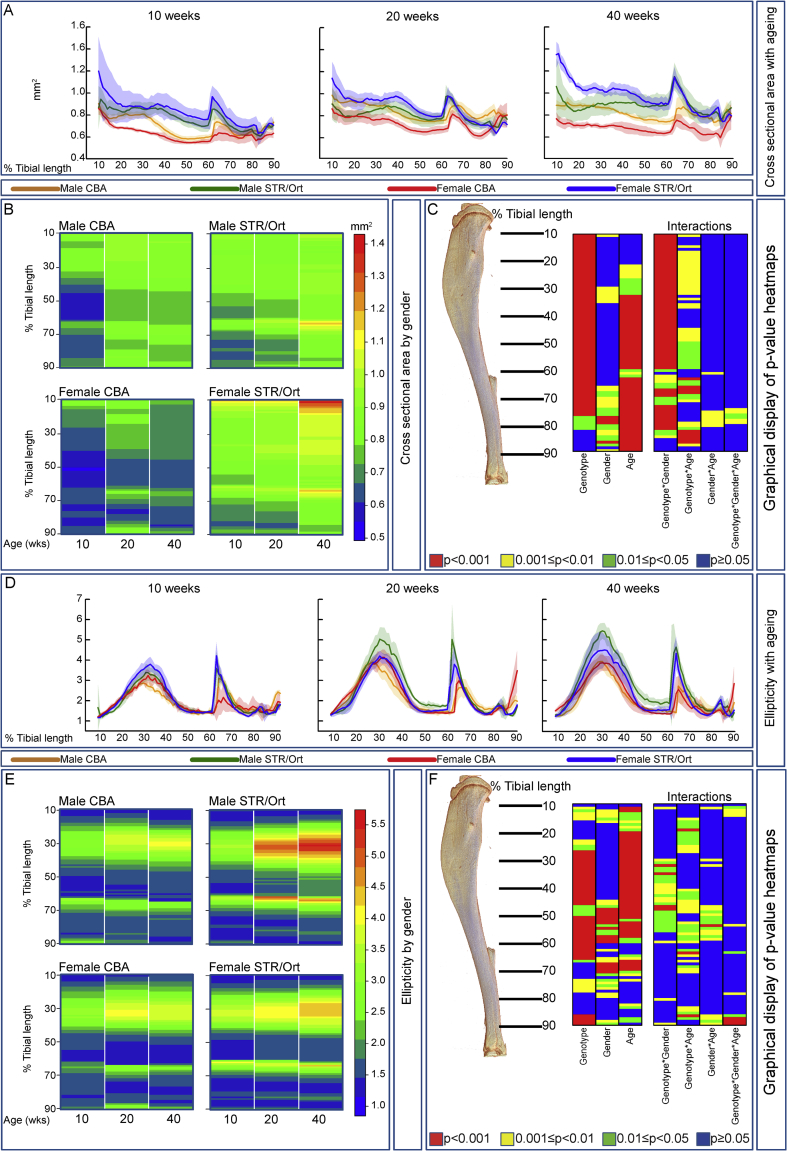
**Analysis of cross sectional area (CSA) and ellipticity along the entire length of the tibia.** (*A*) Mean CSA in male and female CBA (brown and red, respectively) and STR/Ort (green and blue, respectively) mice at 10, 20 and 40 weeks of age. (*B*) ‘Heat map’ representation of identical data-set (red-blue colour scale depicts mean CSA) for male and female CBA (left) and male and female STR/Ort (right) enabling ready comparison (at each percentile of length) between 10, 20 and 40 weeks of age. (*C*) Statistical significance of differences in cross-sectional area along the entire tibial shaft, represented as a heat map. (*D*) Ellipticity in male and female CBA (brown and red, respectively) and STR/Ort (green and blue, respectively) mice at 10, 20 and 40 weeks of age. (*E*) ‘Heat map’ representation of identical data-set (red-blue colour scale depicts average ellipticity) for male and female CBA (left) and male and female STR/Ort (right) in order that comparison can readily be made (at each percentile of length) between 10, 20 and 40 weeks of age. (*F*) Statistical significance of differences in ellipticity along the entire tibial shaft, represented as a heat map. The contributions of genotype (CBA vs STR/Ort), gender, age (10, 20 and 40 weeks), and their interactions at locations from 10 to 90% of tibial length are illustrated. Red *P* < 0.001, yellow 0.001 ≤ *P* < 0.01, green 0.01 ≤ *P* < 0.05 and blue *P* ≥ 0.05. Line graphs represent means with 95% CI. Group sizes were *n* = 5 for male and female CBA and STR/Ort mice.

We therefore examined tibial profiles [Fig. 4(A)] to reveal, consistent with other strains^44^, greater CSA in male than female CBA mice, which is more marked with ageing. Strikingly, STR/Ort mice do not exhibit such trends. Male STR/Ort instead show lower CSA than females at 40 weeks chiefly in proximal regions, with no gender-related divergence distally. We also find that females show uniform and conserved proximodistal CSA patterns in STR/Ort and CBA mice at all ages, and that these deviate markedly in males. Changes in CSA [Fig. 4(B)] emphasise modest age-related increases in tibial CSA in proximal regions in female STR/Ort but in distal regions in male STR/Ort mice. This reveals genotype, age and gender as significant determinants of tibial CSA [Fig. 4(C)].

### STR/Ort mice show distinct gender-related divergence in cortical shape

Examination of shape measures (Supplementary Figs. 2–3) showed significant interaction of genotype and gender for *I*_min_ throughout the entire length and for *I*_max_ mostly in proximal tibia. In addition, significant interactions of genotype, gender and age were detected at many regions. Another shape measure, J (predicts torsion resistance) also showed strong genotype and gender interaction in proximal tibia and an independent influence of age along the entire length; no significant interaction of genotype and age or gender and age were observed (Supplementary Fig. 4). This interaction of genotype and gender exposed higher J proximally in male than female, ageing CBA mice (Supplementary Fig. 4). Intriguingly, J showed dissimilar patterns in STR/Ort, where no gender-related divergence was apparent. Age-related modifications in J were evident in proximal tibia of male and female CBA but were far less marked in both male and female STR/Ort. three-way interactions of genotype, gender and age followed similar pattern to those observed for *I*_min_ and *I*_max_.

Cross-sectional ellipticity was significantly affected by genotype and age (without interaction) along almost the entire tibia (20–90% and 10–60%; Fig. 4(D)–(F)). Profiling revealed most marked age-related increases in ellipticity in proximal tibia of male STR/Ort mice (30–40%; Fig. 4(D)–(F)). Less marked yet similar patterns of age-related ellipticity were observed in male and female CBA and female STR/Ort mice [Fig. 4(D)–(F)]. Minor interactions of genotype, gender and age were detected suggesting that each factor independently contributes to differences in ellipticity.

### Males STR/Ort mice exhibit distinct regional strain and shape (curvature) bias

FEM was performed to predict mechanical environment engendered by axial compressive loading. Lower von Mises stresses (and absolute maximum principal strains) were predicted at the proximal diaphysis (15–45% length) in 10 week-old male STR/Ort compared to both female STR/Ort and CBA mice [Fig. 5(A)–(B)]. Average stresses (and strains) induced at the distal diaphysis (75–100% length) were comparable in all groups. Since such stresses are likely a product of load-induced compressive stress and bending, due to curved morphology, we analysed the tibia moment arm around the loading axis, in the light of CSA. This showed a larger lever arm at the proximal diaphysis in STR/Ort compared with CBA mice [Fig. 5(C)]. However, correspondingly greater CSA at this location in STR/Ort [Fig. 4(A)] lead to lower stresses compared with CBA mice.

**Fig. 5:**
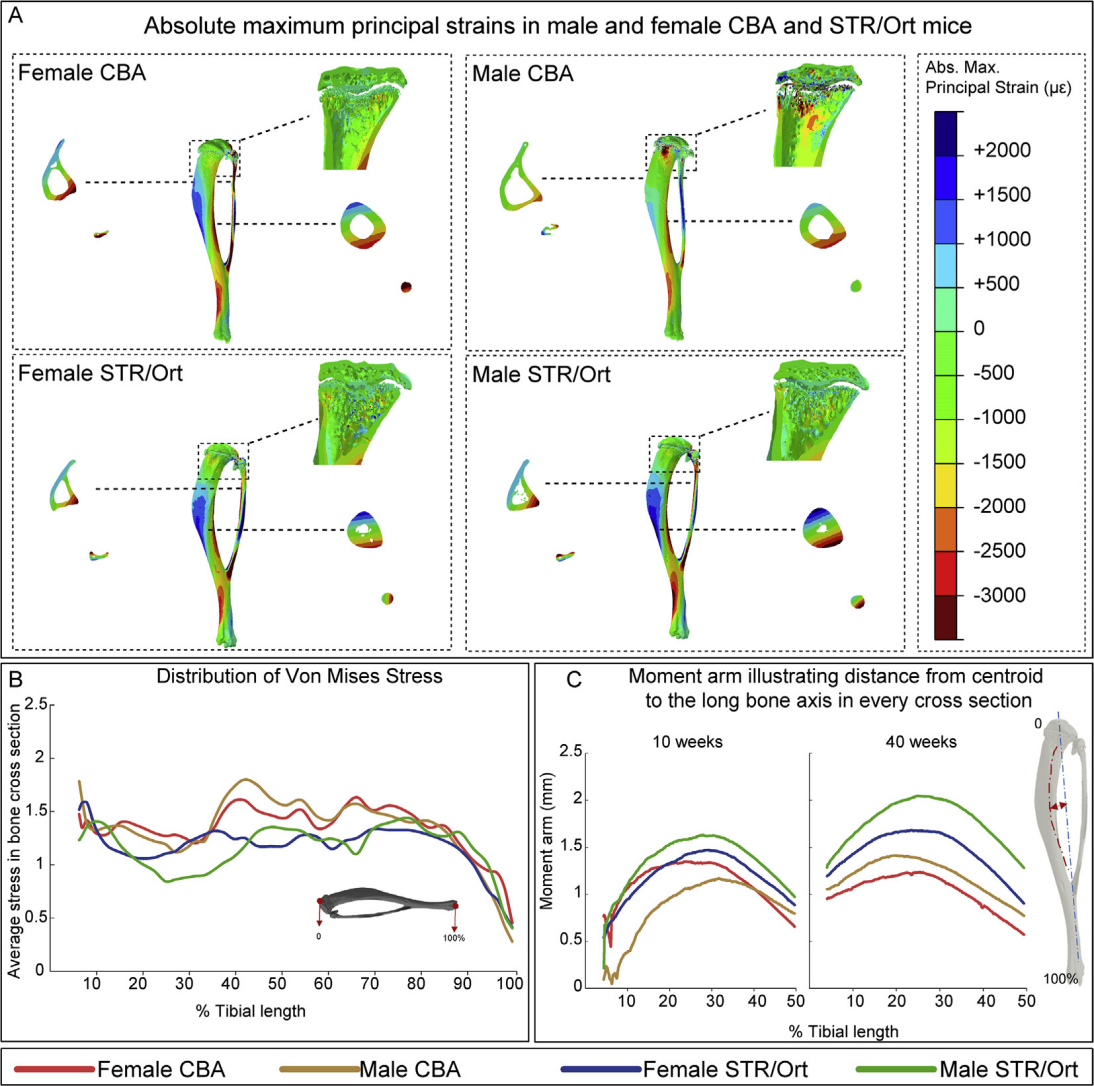
**Analysis of strain distribution along the tibia by finite element modelling (FEM)** **and measurement of tibial curvature.** (*A*) The distribution of principal strain across the tibia in male and female CBA and STR/Ort mice; negative values are compressive and positive values are tensile strains. (*B*) Distribution of Von Mises Stress along the tibia of male and female CBA and STR/Ort mice under bending. (*C*) Curvature lever arm in male and female CBA and STR/Ort mice; calculated as the perpendicular distance from the proximal–distal chord to the centroid at midshaft divided by the radius.

We also found sexually-dimorphic curvature differences, with a greater moment arm in male than in female STR/Ort mice [Fig. 5(C)]. Considering that CSA in 10 week-old mice was similar in both genders [Fig. 4(A)], higher stresses were expected in males; FEM however predicted otherwise. This was explained by further examination of epiphyseal/metaphyseal regions in young growth plate where highly porous epiphysis and greater disconnectedness in male STR/Ort mice is likely responsible for the apparent reduction in load transfer to other regions (Supplementary Fig. 5). We found that greater bone curvature in male STR/Ort was more pronounced by 40 weeks and considering greater CSA, further reduction in load-induced mechanical stress is predicted in ageing male STR/Ort tibiae.

## Discussion

This study identifies bone mass and shape features in male STR/Ort mice that may explain how age and gender interact to predispose to OA. Our studies: (1) confirm HBM phenotype in STR/Ort compared to parental CBA mice; (2) disclose a switch in bones’ sexually-dimorphic behaviour in STR/Ort mice, where trabecular mass, cortical CSA and thickness are unusually all lower in males than females; and (3) reveal that male STR/Ort mice uniquely exhibit greater proximal tibia curvature and ellipticity before OA onset, which become more pronounced.

Profound trabecular HBM has previously been described in STR/Ort mice^29^. Our data confirm trabecular HBM in this OA-prone strain and reveal switching in bones’ sexually-dimorphic phenotype. Exciting data has recently linked HBM to a range of OA risk indicators^11^, including hip OA and osteophyte formation^12^, leading to speculation that greater bone-forming activities predispose to dysplasia and OA risk^45^. This link should be interpreted cautiously, since HBM is also commonly associated with higher body mass index^10^, another potential OA risk factor^46^. Does HBM predispose STR/Ort mice to OA?

Pasold *et al.* (2013) previously observed cortical HBM in STR/Ort mice, which lacked the sexual-dimorphism they observed in trabecular bone. We confirm cortical HBM in STR/Ort but also demonstrate unexpectedly exaggerated, sexually-dimorphic lowering of CSA and cortical thickness in OA-prone male STR/Ort. It remains possible that OA predisposition is underpinned by some hitherto unresolved bone quality difference. Our finding that cortical BMD shows age-related changes in female STR/Ort and both genders of CBA mice, but no such shift during male STR/Ort mouse ageing suggests that these OA-prone mice have greater BMD before OA onset but no further increases with ageing/OA progression. Although difficult to explain, it is tempting to suggest that this links the regulation of bone mass and quality to OA risk.

HBM may contribute a risk but unlikely fully explains OA predisposition in male STR/Ort mice. The fact that we and others observe an amplified HBM phenotype in female STR/Ort, which are protected against OA, suggests that HBM alone is an insufficient explanation. HBM may require an additional, gender-derived OA input, at least in male STR/Ort mice. Strikingly, our curvature and bone cross-sectional ellipticity shape data show sexually-dimorphic differences that are associated with onset and further exaggerated with OA progression only in male STR/Ort, suggesting that cortical shape rather than mass might more fully explain OA in this strain. Lack of similar sexually-dimorphic curvature traits in healthily ageing CBA mice supports this assertion^20^, ^27^, ^28^.

Tibia shape has previously been linked to gait deviations; patients with medial knee OA show altered gait^47^. Gait might be influenced by varus misalignment to increase medial stresses and thus precipitate OA^14^, ^15^, ^48^. Our studies emphasise this link between overall bone shape, gait and OA but are clearly limited by the unpredictable drop-out of STR/Ort mice from the treadmill task. Further studies are therefore required to better test these links.

The divergent tibia shape in male STR/Ort does not, however, translate to predicted resistance to torsion, which instead shows gender-related differences in only CBA mice, and instead male and female STR/Ort mice track closely. It is intriguing that Naruse *et al.*, (2009) previously used gross CT to describe increased tibia torsion in male STR/Ort from 5 to 35 weeks of age^49^ when compared to male C57BL/6 mice. Our data show that predicted resistance to torsion is not, however, dissimilar in male and female STR/Ort, yet males uniquely exhibit greater overall tibia curvature before OA onset, to establish the first, clear sexually-dimorphic link between bone shape and OA predisposition in male mice of this strain. Previous studies had shown gender-related differences in architecture in STR/Ort mice but had failed to explain why OA preferentially targets males^30^. By fully evaluating links between bone architecture and spontaneous OA, our data are the first to offer a sexually-dimorphic link, combining lower than expected cortical bone mass with modified curvature, longitudinally to OA onset and progression. Our use of high resolution CT and analysis of the entire tibia across the ages in STR/Ort and CBA mice of both genders is perhaps pivotal^50^.

We are cognisant that bone shape and gait analysis rely on multiple statistical testing which may, however, introduce limitations. We have reduced multiple testing in our gait evaluations with PCA and circumvented emergence of false positives in cortical bone CT by emphasising only differences encompassing wide regions, where very high levels of statistical significance were reached. We also employed a factorial design to reduce type I errors and increase power of our analyses.

We find that trabecular and cortical bone mass alone, are unlikely to explain OA predisposition. We did however uncover tibia shape features unique to male STR/Ort, with greater curvature and divergent cross-sectional ellipticity compared to all control, non-OA prone mice examined, leading us to hypothesise that bone shape modifications on a HBM background promotes OA. As tibia bone shape measures are rarely reported, it is difficult to test whether this hypothesis applies more generally. Strategies whereby bone shape is modified either by specific regimes of applied loading, by surgery such as high tibia osteotomy or by pharmacological targeting of bone remodelling in animals with HBM phenotypes will allow this proposed causal relationship to joint cartilage integrity and OA to be tested.

## Author contributions

Conception and design: Javaheri, Pitsillides.

Analysis and interpretation of the data: Javaheri, Razi, Piles, De Souza, Chang, Maric-Mur, Hopkinson, Pitsillides.

Drafting of the article: Javaheri, Pitsillides.

Critical revision of the article for important intellectual content: Javaheri, Pitsillides.

Final approval of the article: Javaheri, Lee, Pitsillides.

Provision of study materials or patients: Pitsillides.

Statistical expertise: Piles, Chang.

Obtaining of funding: Lee, Pitsillides.

Administrative, technical, or logistic support: Hopkinson.

Collection and assembly of data: Javaheri, Razi, Piles, De Souza, Chang, Maric-Mur, Hopkinson.

## Conflict of interest

The authors have no conflict of interest to declare.

## Figures and Tables

**Fig. 1a:**
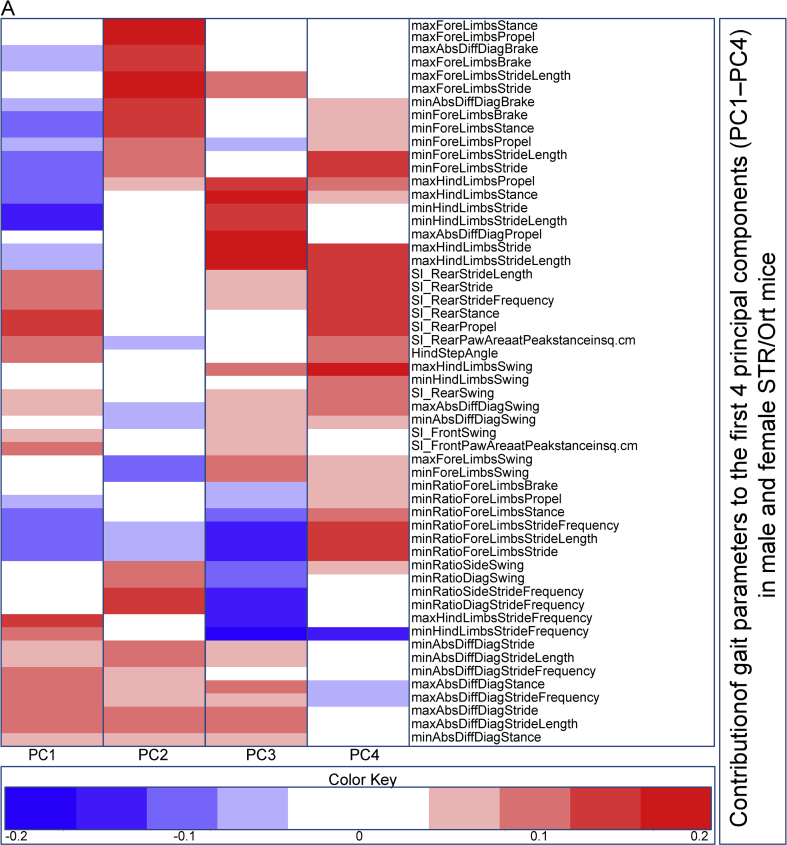
**Sexually dimorphic OA development in STR/Ort mice is linked to longitudinal gait asymmetry.** (*A-B*) Heatmaps representing the contribution of the gait parameters to the first 4 principal components (PC1–PC4) in male and female STR/Ort mice.

**Fig. 1b:**
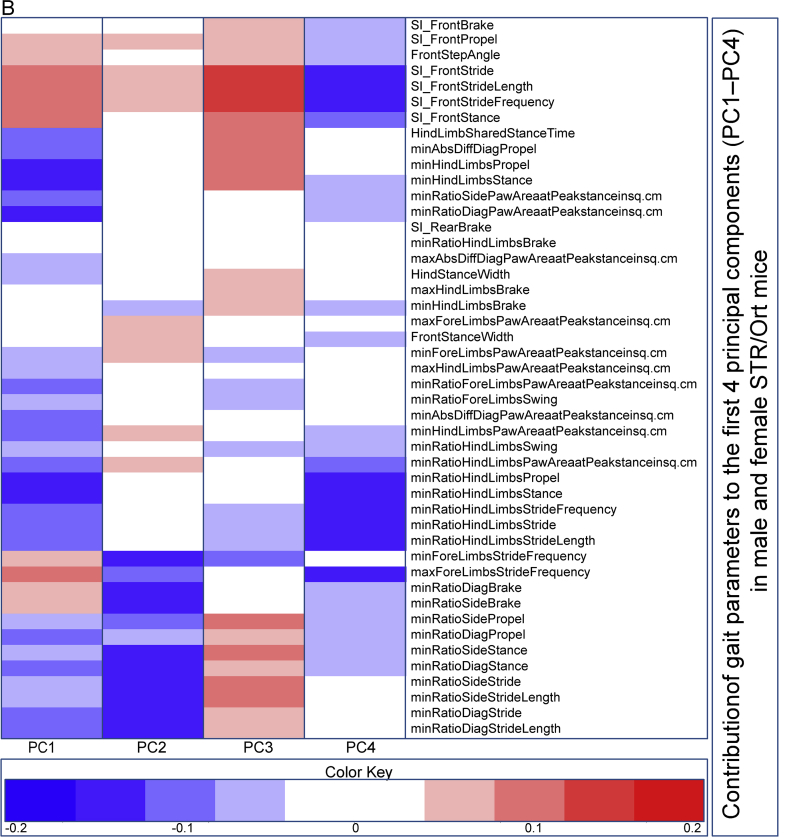
**Sexually dimorphic OA development in STR/Ort mice is linked to longitudinal gait asymmetry.** (*A-B*) Heatmaps representing the contribution of the gait parameters to the first 4 principal components (PC1–PC4) in male and female STR/Ort mice

**Fig. 2:**
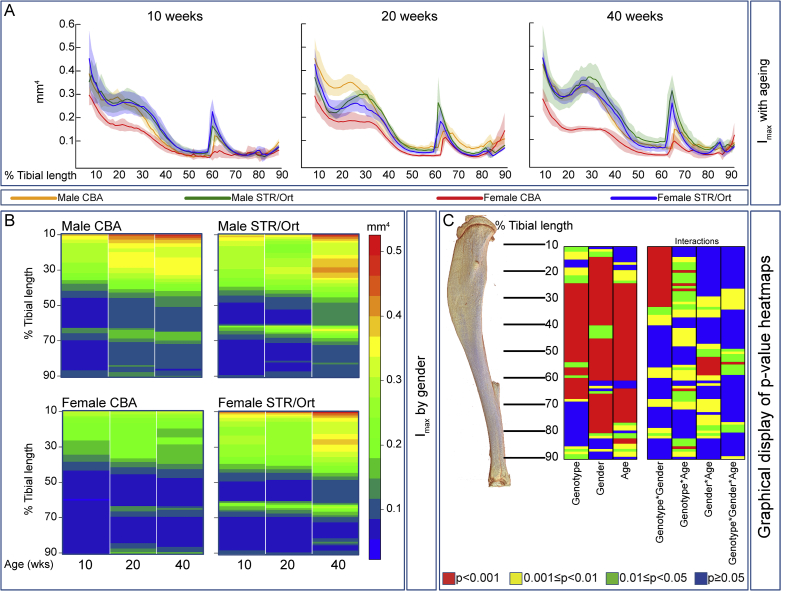
**Analysis of *I***_**max**_**along the entire length of the tibia.** (*A*) Mean *I*_max_ in male and female CBA (brown and red, respectively) and STR/Ort (green and blue, respectively) mice at 10, 20 and 40 weeks of age. (*B*) ‘Heat map’ representation of identical data-set (red-blue colour scale depicts mean *I*_max_) for male and female CBA (left) and male and female STR/Ort (right) enabling ready comparison (at each percentile of length) between 10, 20 and 40 weeks of age. (*C*) Statistical significance of differences in *I*_max_ along the entire tibial shaft, represented as a heat map. The contributions of genotype (CBA vs STR/Ort), gender, age (10, 20 and 40 weeks), and their interactions at locations from 10 to 90% of tibial length are illustrated. Red *P* < 0.001, yellow 0.001 ≤ *P* < 0.01, green 0.01 ≤ *P* < 0.05 and blue *P* ≥ 0.05. Line graphs represent means with 95% CI. Group sizes were *n* = 5 for male and female CBA and STR/Ort mice.

**Fig. 3:**
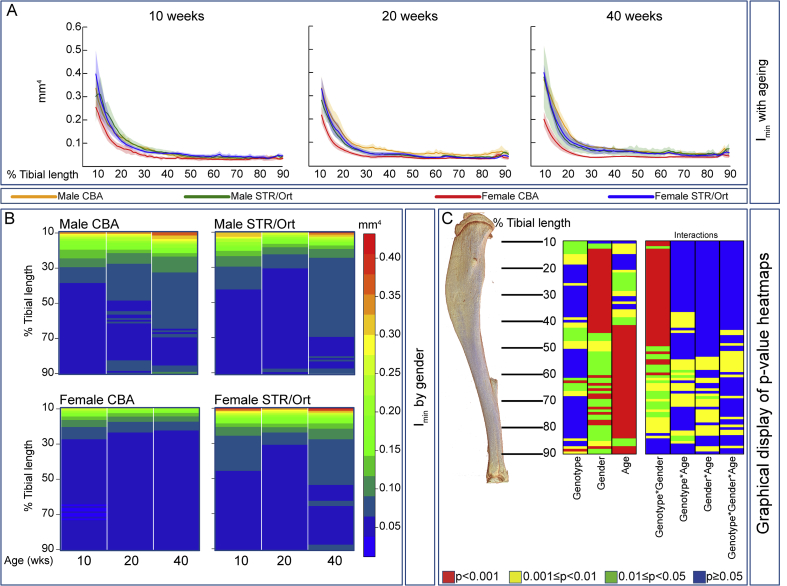
**Analysis of *I***_**min**_**along the entire length of the tibia.** (*A*) Mean *I*_min_ in male and female CBA (brown and red, respectively) and STR/Ort (green and blue, respectively) mice at 10, 20 and 40 weeks of age. (*B*) ‘Heat map’ representation of identical data-set (red-blue colour scale depicts mean *I*_min_) for male and female CBA (left) and male and female STR/Ort (right) enabling ready comparison (at each percentile of length) between 10, 20 and 40 weeks of age. (*C*) Statistical significance of differences in *I*_min_ along the entire tibial shaft, represented as a heat map. The contributions of genotype (CBA vs STR/Ort), gender, age (10, 20 and 40 weeks), and their interactions at locations from 10 to 90% of tibial length are illustrated. Red *P* < 0.001, yellow 0.001 ≤ *P* < 0.01, green 0.01 ≤ *P* < 0.05 and blue *P* ≥ 0.05. Line graphs represent means with 95% CI. Group sizes were *n* = 5 for male and female CBA and STR/Ort mice.

**Fig. 4:**
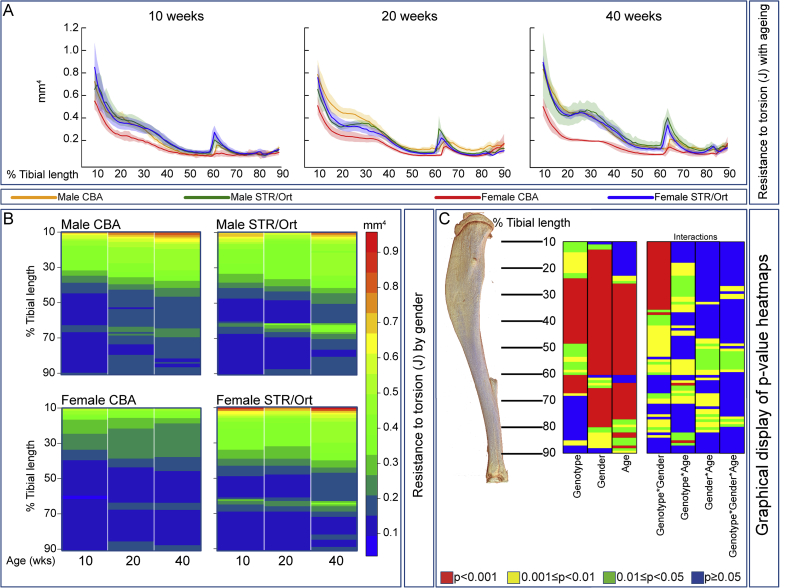
**Analysis of J (measure of predicted resistance to torsion) along the entire length of the tibia.** (*A*) J in male and female CBA (brown and red, respectively) and STR/Ort (green and blue, respectively) mice at 10, 20 and 40 weeks of age. (*B*) ‘Heat map’ representation of identical data-set (red-blue colour scale depicts average ellipticity) for male and female CBA (left) and male and female STR/Ort (right) enabling ready comparison (at each percentile of length) between 10, 20 and 40 weeks of age. (*C*) Statistical significance of differences in J (measure of predicted resistance to torsion) along the entire tibial shaft, represented as a heat map. The contributions of genotype (CBA vs STR/Ort), gender, age (10, 20 and 40 weeks), and their interactions at locations from 10 to 90% of tibial length are illustrated. Red *P* < 0.001, yellow 0.001 ≤ *P* < 0.01, green 0.01 ≤ *P* < 0.05 and blue *P* ≥ 0.05. Line graphs represent means with 95% CI. Group sizes were *n* = 5 for male and female CBA and STR/Ort mice.

**Fig. 5:**
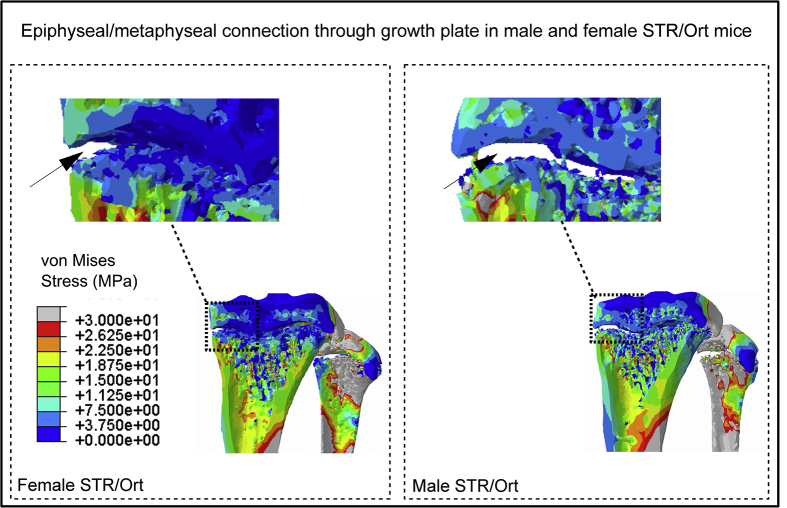
Epiphyseal/metaphyseal region of 10-week old male and female STR/Ort mice showing von Misses stress and the extent of growth plate connectivity.

## Appendix A Supplementary data

Table I Whole body weight of male and female CBA and STR/Ort mice at 10, 20 and 40 weeks of age.

Table II Parameter estimates (95% confidence interval) of the fixed effects and variances for the random effects and residual for the first 4 gait principal components using linear mixed effects models.
