# Supplementary material for: Sexually dimorphic tibia shape is linked to natural osteoarthritis in STR/Ort mice
Source: Osteoarthritis Cartilage. 2018 Jun;26(6):807–17. doi: 10.1016/j.joca.2018.03.008 (PMC5987380; doi:10.1016/j.joca.2018.03.008)
Supplement: Table I — Whole body weight of male and female CBA and STR/Ort mice at 10, 20 and 40 weeks of age. [file mmc1.docx]

| **Weight (g)** | **10 weeks** | | **20 weeks** | | **40 weeks** | |
| --- | --- | --- | --- | --- | --- | --- |
|  | Mean | CI 95% | Mean | CI 95% | Mean | CI 95% |
| Female CBA | 16.6 | 12.47 – 20.77 | 22.6 | 20.79 – 24.37 | 29.1 | 26.92 – 31.24 |
| Male CBA | 24.2 | 20.09 – 28.39 | 28.8 | 23.73 – 33.91 | 33.36 | 29.7 – 37.02 |
| Female STR/Ort | 19.6 | 16.25 – 22.95 | 29.2 | 24.6 – 33.8 | 38 | 36.04 – 39.96 |
| Male STR/Ort | 28.3 | 22.81 – 33.75 | 36.4 | 31.7 – 41.1 | 44.6 | 40.2 – 49.5 |

Table 1

| **Parameter** | **Age** | **Genotype** | **Gender** | **Age * Genotype** | **Age * Gender** | **Genotype * Gender** | **Age * Genotype * Gender** |
| --- | --- | --- | --- | --- | --- | --- | --- |
| **Bone length (mm)** | ≤0.001 | ≤0.05 | NS | NS | NS | NS | NS |
| **Trabecular** |  |  |  |  |  |  |  |
| Percent bone volume  (%) | NS | ≤0.001 | ≤0.01 | NS | NS | ≤0.05 | NS |
| Trabecular number (mm^-1^) | ≤0.001 | ≤0.001 | ≤0.05 | NS | ≤0.05 | ≤0.001 | NS |
| Trabecular thickness (mm) | ≤0.001 | NS | ≤0.01 | ≤0.05 | NS | NS | ≤0.05 |
| Trabecular separation (mm^-1^) | ≤0.001 | ≤0.001 | NS | ≤0.001 | NS | NS | NS |
| Degree of anisotropy | ≤0.001 | ≤0.01 | ≤0.05 | ≤0.05 | NS | NS | NS |
| **Cortical** |  |  |  |  |  |  |  |
| Cortical BMD (g.cm^-3^) | ≤0.001 | NS | ≤0.01 | ≤0.001 | NS | ≤0.05 | ≤0.001 |
| Total porosity (%) | <0.05 | ≤0.001 | NS | <0.05 | NS | <0.01 | NS |

Table 2
