# Supplementary material for: Sexually dimorphic tibia shape is linked to natural osteoarthritis in STR/Ort mice
Source: Osteoarthritis Cartilage. 2018 Jun;26(6):807–17. doi: 10.1016/j.joca.2018.03.008 (PMC5987380; doi:10.1016/j.joca.2018.03.008)
Supplement: Table II — Parameter estimates (95% confidence interval) of the fixed effects and variances for the random effects and residual for the first 4 gait principal components using linear mixed effects models. [file mmc2.docx]

| **Estimate** | **PC1** | **PC2** | **PC3** | **PC4** |
| --- | --- | --- | --- | --- |
| **Fixed effects** | | | | |
| Intercept | 5.055(-2.104, 12.214) | -0.384 (-5.866, 5.097) | **-8.347 (-12.768, -3.925)** | -2.631 (-6.453, 1.191) |
| Gender (Male) | **-16.269 (-27.281, -5.259)** | 2.762 (-5.814, 11.338) | -4.690 (-11.589, 2.209) | -2.654 (-8.547, 3.238) |
| Age (month) | -1.293 (-4.413, 1.827) | -0.251 (-2.658, 2.156) | **3.149 (1.204, 5.096)** | 0.746 (-0.925, 2.418) |
| Age^2^ (month^2^) | 0.003 (-0.324, 0.329) | 0.061 (-0.196, 0.318) | **-0.257 (-0.462, -0.052)** | -0.056 (-0.231, 0.119) |
| Male*Age | **6.838 (1.842, 11.834)** | -1.371 (-5.313, 2.572) | 2.873 (-0.296, 6.042) | 2.006 (-0.675, 4.686) |
| Male*Age^2^ | **-0.646 (-1.195, -0.097)** | 0.214 (-0.229, 0.657) | **-0.413 (-0.764, -0.061)** | -0.255 (-0.550, 0.039) |
| **Variances for random effects** | | | | |
| Mouse | 1.877 | 1.422 | 0.000 | 0.346 |
| Age within Mouse | 0.000 | 0.000 | 0.081 | 0.000 |
| Age^2^ within Mouse | 0.000 | 0.003 | 0.000 | 0.000 |
| **Residual variance** | 21.105 | 11.742 | 8.079 | 6.052 |

Table 2
